# Report of Clinic, from St. John’s Hospital

**Published:** 1880-08-20

**Authors:** J. Emory Lamphear


### REPORT OF CLINICS.

### FROM CLINIC OF PROF. J. P. KINGSLEY AT ST. JOHN'S HOSPITAL.

Reported by J. Emory Lamphear.

April 17th.—Freide H——, aged five years. Has been complaining for about four years; has been under the treatment of various physicians, but without relief. Complains of an intolerable itching around the anus; for a long time has been vomiting after meals, the ejections being acid in character; has slight fever every morning; appetite poor, but she appears quite well nourished.

Diagnosis: Pruritus ani and indigestion. Treatment:

Internally.

℞ Bismuthi subnit............ ʒ ij.

Syrupi simplicis............ f℥ j.

Aquæ cinnamoni............ f℥ j.

M. S. Teaspoonful three times daily.

Locally.

℞ Acidi carbolici............ gtt. x.
Empl. plumbi............ ℥ ss.
Ungt. petrolei............ ℥ ss.

M. S. Apply.

April 30th. Better than she has been since commencement of affection. Itching entirely allayed.

This case is one of interest, inasmuch as it illustrates the effect of a few simple remedies, when the nature of the case is thoroughly understood and the proper remedies selected.

In relieving the indigestion by the administration of the bismuth, one great point in the treatment was accomplished, namely, the removal of a cause tending to keep up the irritation; and by the use of the carbolic acid the intense itching was allayed until the source of the irritation could be removed. Others had treated the case by local applications alone, which could result at best in but a temporary relief, while combined with the internal treatment, a successful termination was the result.

March 24th.—Katie F——, aged four months. Had a slight cough for about three weeks. During that time has been troubled by persistent diarrhœa, but unaccompanied by any marked febrile action. Vomits; very cross and restless. The discharges from the bowels very slimy, but no appearance of blood; acid in character; contain undigested milk.

Diagnosis: Entero-colitis. Treatment:

℞ Sodii bicarbonatis............ ℈ j.

Aquæ cinnamomi............ f℥ ij.

M. S. Teaspoonful every three hours.

March 29.—Diarrhœa entirely disappeared.

These cases of intestinal catarrh in infants are of very frequent occurrence, especially during the summer months. Although the affection may arise from a variety of causes, yet in the greater proportion of cases, it may be traced to some error in diet or to indigestion; if due to the former, a removal of the cause will often suffice, while if it proceeds from indigestion, the administration of some simple remedy will often effect a cure. In this case the disturbance evidently proceeded from indigestion, as is proven by reference to the history; moreover, the matter vomitted was acid in nature, as were the passages, hence an indication of an antacid. So, instead of giving opiates, astringent preparations, etc., as is so often done in these cases, a little bicarbonate of soda was administered. And what was the result? Five days later the child was reported well, in better health than it had enjoyed for some time.

February 3d.—Harriet S——, aged four years. Three months ago she became paralyzed in both lower extremities; could move left leg a little; complete paralysis of right. Suffered great pain in the limbs for two weeks; since pain subsided, electricity has been employed with great benefit. Can now stand upon left leg.

Diagnosis: Infantile paralysis. Treatment: Faradaic current locally.

Internally:

| | | |
|---|---|---|
| ℞ | **Strychniæ sulph** | gr. j. |
| | **Ferri pyrophosphat** | ʒj. |
| | Syr. aurantii | f℥ iij. |
| | Aquæ cinnamomi | f℥ iij. |

M. S. Teaspoonful three times daily.

April 5th.—Has been coming regularly twice a week and is very much improved. The left leg which was also paralyzed, is now completely restored, and the right foot was moved voluntarily yesterday.

April 19th.—Recovering quite rapidly. Can now walk about quite well.

During the first two or three seances the muscular contractions were marked upon the application of the interrupted current, but after that the response was very feeble—almost imperceptible; yet had the continuous current been applied, the contractions would still have been noticed; for it is the rule that in all cases where recovery is possible, the muscles retain their contractility when the continuous current is employed.

This paralysis made its appearance during a severe attack of intermittent fever; the child was confined to the bed for several days, and it was only when it attempted to arise that the paralysis was discovered. No cause could be assigned for its occurrence, and this is usually the case in true infantile paralysis. Neither is there much more positive knowledge in regard to the part of the nervous system which is the seat of the lesion giving rise to the paralytic disturbance.

The treatment which was instituted in this case, is that from which, as experience has proven, the most benefit can be derived.—*St. Louis Courier of Medicine.*

